# Psychological Health of Deaf Pre-Teens and Teenagers with Cochlear Implants and Maternal Psychological Features: A Pilot Study

**DOI:** 10.3390/healthcare13050498

**Published:** 2025-02-25

**Authors:** Valeria Caragli, Michela Camia, Maristella Scorza, Elisabetta Genovese, Antonio Maria Persico, Paola Benincasa, Erika Benassi

**Affiliations:** 1 Otorhinolaryngology-Head and Neck Surgery, Audiology Program, University of Modena and Reggio Emilia, 41125 Modena, Italy; valeria.caragli@unimore.it; 2 Department of Biomedical, Metabolic and Neural Sciences, University of Modena and Reggio Emilia, 41125 Modena, Italy; michela.camia@unimore.it (M.C.); maristella.scorza@unimore.it (M.S.); 3 Audiology Program, Department of Medical and Surgical Sciences for Children and Adults, University of Modena and Reggio Emilia, 41125 Modena, Italy; elisabetta.genovese@unimore.it; 4 Child and Adolescent Neuropsychiatry Program, Modena University Hospital & Department of Biomedical, Metabolic and Neural Sciences, University of Modena and Reggio Emilia, 41125 Modena, Italy; antonio.persico@unimore.it; 5 ENT Unit, Ramazzini Hospital, Carpi AUSL, 41012 Carpi, Italy; p.benincasa@ausl.mo.it; 6 Department of Education and Humanities, University of Modena and Reggio Emilia, 42121 Reggio Emilia, Italy

**Keywords:** deafness, cochlear implant, teenagers, mothers, mental health, resilience, sharing emotions

## Abstract

Background/Objectives: The psychological health of deaf children and adolescents with cochlear implants (CIs) appears to be related to the degree of auditory and linguistic recovery achieved, as well as contextual factors. Few studies have investigated the influence that maternal psychological characteristics and resources may have in supporting the mental health of these children and adolescents. The aim of this pilot study was to investigate the psychological well-being of pre-teens/teenagers with CIs and the mental health of their mothers. The secondary aim was to analyze which maternal characteristics (anxiety, depression, resilience, and time spent sharing emotions) were most related to the psychological health of the pre-teen/teenager. Methods: A group of 15 pre-teens/teenagers with CIs and 27 hearing peers and their mothers participated in the study. The Strengths and Difficulties Questionnaire, the Generalized Anxiety Disorder Scale, the Beck Depression Inventory II, the Connor–Davidson Resilience Scale, and an additional ad hoc question quantifying the time that the mothers dedicate to conversing with their sons/daughters about the emotions were administered to the included subjects. Results: No significant differences between the two groups of pre-teens/teenagers emerged; however, a great percentage of pre-teens/teenagers with CIs appeared at higher risk for developing psychopathology. The resilience scores for both groups of mothers were lower than anticipated and related to the psychological health of pre-teens/teenagers. Conclusions: These results underscore the need for targeted psychological support alongside auditory rehabilitation and suggest avenues for enhancing family-centered care in this clinical population.

## 1. Introduction

Permanent childhood deafness is the most common sensory disorder at birth. According to the World Health Organization [1], children and adolescents who are Deaf or Hard of Hearing (DHH) constitute 2–5 out of 1000 live births every year, with a total of 34 million children and adolescents worldwide.

Approximately 5–10% of deaf children are born to families in which at least one parent is deaf and utilizes sign language [2]. Sign languages are recognized as natural languages [3] and have contributed to a paradigm shift in the perception of individuals with DHH from a disability-focused view to a socio-cultural perspective [4]. Research indicates that deaf signing children achieve language milestones as rapidly as their hearing peers [5], with positive effects on their emotional well-being and quality of life (QoL). Conversely, 90–95% of deaf children who are born to hearing parents typically follow the path of amplification and verbal language learning. In some cases, these children also learn sign language from hearing parents or non-native signers. As a result, DHH children are at greater risk of developing language and learning process delays than normal-hearing children [5,6,7,8].

It is well known that the presence of deafness may impact the QoL and psychological well-being of families composed of hearing parents and DHH children [9].

Conflicting results emerge from the analysis of literature concerning the risk of psychopathology development in deaf pre-teens/teenagers with cochlear implants (CIs). On the one hand, deaf pre-teens/teenagers with CIs present a lower QoL and more psychological difficulties when compared to normal-hearing peers [10]. Moreover, DDH patients may have lower academic performance [11] and limited executive functions (such as working memory, attention, or inhibition/flexibility) [12,13], experiencing feelings of loneliness, social isolation, and frustration [14]. Consequently, psychological disorders, altered emotional responses, and inappropriate behavioral reactions in social interactions may occur [9,10]. On the other hand, there are children and adolescents with CIs who do not present difficulties or mental ill health compared to normal-hearing peers and have good social networks [15]. Interestingly, it has been demonstrated that while children with CIs aged 8–12 experience a lower QoL compared to hearing children peers, adolescents with CIs aged 13–16 feel the same as normal-hearing peers [9]. Nonetheless, there is still a lack of studies focusing on the psychological health of DHH adolescents in different social contexts. One of the hypotheses as to why psychological diseases may occur has been attributed to the presence and use of assistive technology (AT). However, the results indicated a positive psychosocial impact across all device types, particularly for Video Relay Services (VRS), Hearing Aids (HAs), and CIs, which showed better outcomes compared to common Information and Communication Technologies (ICTs). Specifically, hearing aids were found to enhance quality of life, self-esteem, and social well-being, highlighting that self-esteem is a critical factor that influences the abandonment of HAs and CIs. However, it should be noted that a significant percentage (20%) of CI users seem to abandon their devices, with no strong correlations between abandonment and demographic variables or time since implantation [16].

Conflicting findings also seem to emerge with regard to parents’ psychological well-being, especially in hearing parents with deaf children. Deaf parents often possess a strong connection to deaf culture, which can positively influence their sense of identity and community, thereby enhancing their psychological well-being [17]. Effective communication within the family, primarily facilitated through sign language, promotes emotional closeness and minimizes misunderstandings. Access to supportive networks, such as deaf communities and organizations, provides valuable resources and emotional support, positively contributing to the mental health of deaf parents [17]. Conversely, a different scenario unfolds for hearing parents with a deaf child or adolescent. In this regard, to belong to the deaf community and to have a deaf child is almost a “relief” for deaf parents, while hearing parents who have a deaf child may experience a significant challenge when facing the condition and accepting the diagnosis, thus impacting not only communicative exchanges but also QoL and daily performances. Hearing parents with DHH children and adolescents may struggle with son/daughter outcomes and communication skills [18] and may be exposed to higher and more frequent levels of stress, in terms of both practical and emotional involvement [19,20], facing complex situations or difficult decisions [21]. Risk factors for general stress usually include the presence of other conditions associated with hearing loss, such as cognitive delays, congenital infections, and low levels of parental education [22]. Nonetheless, in some cases, good outcomes may be achieved in similar situations [23,24]. The decision to undergo CI surgery appears to decrease parenting stress, potentially due to the greater knowledge or the parents’ belief that they have done everything possible for their child [23]. Mothers of children with CI present with greater general health in terms of anxiety, depression, and social dysfunction [25]. This result appears to be related to the auditory, language, and social improvements of their children, which makes feel them more comfortable and safer in social environments, as well as less stressed when dealing with challenges [25]. Parents of deaf children express satisfaction with their overall quality of life and have a positive view of the interests, recreational activities, and physical health of their children [26]. A high level of education in the mother and a cooperative family environment also seem to be protective factors against stress [27].

Although the impact of both the child’s clinical conditions and social and environmental characteristics on the mother’s psychological health has been extensively investigated, there is a lack of knowledge about the impact of stress-coping factors, such as resilience, on children and adolescent’s outcomes with CIs [23]. In this regard, resilience may represent a protective factor against stress and psychopathology [28]. The mother’s resilience is described as the ability to cultivate strength and recover, gain strength, or bounce back to an acceptable level of personal and family functioning after the stressor of the diagnosis of a chronic health condition in a child. Families who demonstrate higher levels of resilience tend to score higher on measures of well-being and QoL. This indicates that high levels of resilience lead to a person or family feeling more satisfied with their lives. Allen [29] found that the level of maternal resilience influences how these families react to and move forward with the diagnosis of childhood deafness. Similarly, Ahlert and Greeff [30] reported that some family system qualities associated with resilience enhance family functioning and their adaptation to the diagnosis of deafness. Zhang and colleagues [31] highlighted that resilience mediates the effect of stress on coping strategies in a group of 231 parents of children with CIs.

Moreover, the mother’s resilience appears to be related to the emotional health of the child with a disability too. Children are well attuned to their mother’s emotional states, and the mother’s concerns regarding the implications of a disability might compromise the mother’s ability to sensitively recognize and respond to her children’s cues or distress [32]. Conversely, parental communication focused on emotions, emotional atonement, and positive emotional directives are associated with better social and emotional competence of their child [33]. Thus, maternal resilience and response to their adolescents’ emotions and concerns seem to influence the psychological outcomes of adolescents, especially in the presence of disabilities [34,35]. However, studies on these topics are still scant.

This pilot study first aims to investigate the psychological well-being of deaf pre-teens and teenagers with CIs and without associated disabilities or developmental disorders. Specifically, we address deaf pre-teens and teenagers with very good auditory recovery and good linguistic and learning performance, with the aim of examining whether, despite this good recovery, they still exhibit psychological difficulties. Moreover, we focus on patients’ mothers with the purpose of measuring both their psychopathological symptoms and their positive psychological features, i.e., resilience and frequency of communication focused on emotions, named “time for sharing emotions”.

## 2. Materials and Methods

### 2.1. Participants

A total of 42 pre-teens/teenagers and their mothers were enrolled: 15 of them were pre-teens/teenagers with DHH and CIs (deaf group), while 27 were hearing pre-teens/teenagers (control group).

Patients with CIs were recruited at the Audiology Unit of the Modena University hospital and at the Hospital of Carpi, Northern Italy. The inclusion criteria were (a) CI surgery before 36 months; (b) the absence of other sensorimotor and neurological impairment or developmental disorders (no language and/or learning disorders are reported); (c) hearing parents and Italian as native/dominant language; (d) exposure to oral communication before and after implantation (non-use of sign language); (e) CI activation within one month after the surgery; (f) continued use of the device; and (g) Pure Tone Average (PTA) after CI > 25–30 dB/HL (which reflects a very good auditory recovery). Three patients wore a hearing aid on the unimplanted ear (bimodal stimulation) and six of them had bilateral CIs. The mean age of the pre-teens/teenagers with CIs was 13.16 (SD = 2.27; range 10.42–16.83) and the group included 8 males and 7 females. Their mothers’ mean age was 43.53 years (SD = 5.22; range 37–54). Further details of the deaf group are reported in Table 1.

The 27 hearing pre-teens/teenagers (mean age = 13.40; SD = 1.21; range 11.67–16.83; 11 males and 16 females) were recruited from Modena (Italy) schools that voluntarily participated in the study. To be enrolled, the following criteria had to be met: (a) Italian as the native/dominant language and (b) absence of sensorimotor and neurological impairment or developmental disorders. Their mothers’ mean age was 46.96 years (SD = 5.26; range 33–56). Mothers with disabilities or psychiatric disorders were excluded in both groups.

There were no significant differences between the two groups of pre-teens/teenagers regarding age [*t* (40) = −0.379; *p* = 0.709] and gender [*χ*2 (1, *N* = 42) = 0.617, *p* = 0.432]. The two groups of mothers slightly differed in age [*t* (40) = −2.030; *p* = 0.049] but not in the level of education [*χ*2 (1, *N* = 42) = 0.858, *p* = 0.354].

### 2.2. Procedure

A speech therapist selected the pre-teens/teenagers according to the inclusion criteria. The families were contacted by the researcher and invited to participate in the study. Out of 24, 15 families agreed to participate. Regarding the mothers of hearing pre-teens/teenagers, an invitation letter was distributed in several schools in the city of Modena. Approximately 50 families responded and 27 of them met the inclusion criteria.

An online survey was administered to all mothers from 1 May to 30 June 2022. The link to the online survey was distributed by the experimenter and school directors to the mothers of deaf and normal-hearing pre-teens/teenagers, respectively. Parents of participants were informed about the purpose of the study, the voluntary nature of their participation, and their right to withdraw at any time. Subsequently, parents provided their informed consent to participate in the study. The survey comprised an initial section in which mothers answered general questions about socio-demographic characteristics (such as age and education), as well as questions regarding their child. When focusing on their child, mothers were asked to complete the Strengths and Difficulties Questionnaire (SDQ) [36]. Turning their attention to themselves, the mothers were required to complete the 7-item Generalized Anxiety Disorder Scale (GAD-7) [37], the Beck Depression Inventory II (BDI-II) [38,39], and the Connor–Davidson Resilience Scale (CD-RISC 25) [40]. An ad hoc question aimed at quantifying the time that the mothers dedicate to conversing with their sons/daughters about the emotions felt was also asked. The survey took about 15 min to be completed. The study met the ethical guidelines for human subject protection, including adherence to the legal requirements of the country (Declaration of Helsinki), and it received formal approval from the local research Ethical Committee of the University of Modena and Reggio Emilia (protocol code 0000871/22 on 12 January 2022).

### 2.3. Standardized Measures

Strengths and Difficulties Questionnaire (SDQ) [36,41]. The questionnaire consists of 25 items assessing the psychological health of children and adolescents. It evaluates emotional, behavioral, attentional, and social difficulties (four subscales), as well as strengths (one subscale), from the perspectives of the children/adolescents themselves, their parents, and their teachers. For the purposes of our study, we used the parent’s version and only the four subscales for psychological difficulties. The four subscales for the child’s psychological difficulties concern emotional symptoms (items 3, 8, 13, 16, and 24), conduct problems (items 5, 7, 12, 18, and 22), hyperactivity (items 2, 10, 15, 21, and 25), and peer relationship problems (items 6, 11, 14, 19, and 23). The strengths subscale concerns prosocial behaviors (items 1, 4, 9, 17, and 20). Each item is scored on a 3-point Likert scale: 0 “not true”, 1 “true”, or 2 “absolutely true”. Items 7, 11, 14, 21, and 25 must be inverted for the correct interpretation of the results. The four SDQ subscales for the child’s difficulties are grouped into the total difficulties score, with higher scores reflecting a worse psychological condition. The total score may be categorized into three psychopathology degrees: not psychopathology (0–13), slight risk of psychopathology (14–16), and high risk of psychopathology (17–40). The SDQ demonstrated adequate psychometric properties [41].

Generalized Anxiety Disorder Scale (GAD-7) [37]. The GAD-7 consists of seven items that assess core symptoms of generalized anxiety and inquire about the frequency with which respondents have experienced these symptoms over the past two weeks. Respondents rate the severity of their symptoms using a 4-point Likert scale, ranging from 0 (not at all) to 3 (almost every day), resulting in a total score between 0 and 21. This score is then categorized into four levels of anxiety: normal (0–4), mild (5–9), moderate (10–14), and severe (15–21). The GAD-7 is a highly validated screening tool, demonstrating excellent internal consistency (Cronbach’s alpha = 0.911).

Beck Depression Inventory II (BDI-II) [38,39]. The BDI-II is a commonly used self-report questionnaire designed to assess depressive symptoms in both adolescents and adults. It comprises 21 items that evaluate the presence and severity of depression. Respondents select one of four possible answers for each item. Participants are instructed to answer each question based on their experiences over the past two weeks. The total score ranges from 0 to 63. Based on the obtained score, the individual’s condition can be classified as normal (0–13), mild depression (14–19), moderate depression (20–29), and severe depression (30–63). The Italian version of the BDI-II is comparable to the original edition with a Cronbach alpha of 0.86 for the mental factors and a Cronbach alpha of 0.65 for the somatic factors.

Connor–Davidson Resilience Scale (CD-RISC 25) [40]. The CD-RISC 25 is a standardized self-report scale designed to assess resilience in adults. It consists of 25 items, each with a 5-point response scale: not true at all (0), rarely true (1), sometimes true (2), often true (3), and true nearly all of the time (4). Aligned with the “3 Cs” theoretical framework of psychological resilience, the items focus on control (e.g., “I am able to adapt to change”), coherence (e.g., “Having to face stress has made me stronger”), and connectedness (e.g., “There is someone in my life who is capable of helping me in times of need”). Respondents rate the scale based on their experiences over the past month. The total score ranges from 0 to 100, with higher scores indicating greater resilience. The CD-RISC 25 has been validated in both general and clinical populations, demonstrating strong psychometric properties.

### 2.4. Time for Sharing Emotions

With the aim of quantifying the time that the mother dedicates to conversing with her son/daughter about the emotions he/she is experiencing, an ad hoc question was formulated, namely, “How often do I take time to reflect with my son/daughter on the emotions he/she has experienced during the day or in the recent period?”. An in-depth explanation was provided to mothers in the questionnaire. Specifically, we indicated that the question did not mean simply asking “how are you?” or “how did it go today?”, but rather it referred to how often she spoke to her son/daughter about the emotions felt in different circumstances, e.g., “How did you feel at school today?” “Why did you feel that way?”. We asked the mothers to respond by referring to the last 6 months and in terms of the average frequency. Mothers could respond on an ordinal scale from 0 to 10, where 0 corresponded to “almost never” and 10 corresponded to “every day of the week, for at least half an hour”. With this question, it was thus possible to detect the frequency of this behavior rather than its mere presence/absence. For details on the response scale, see Table 2.

### 2.5. Data Analyses

All statistical analyses were carried out using SPSS 23.0 for Windows, with an alpha level of 0.05. Prior to conducting analyses, the data were checked for violations of assumptions of normality and homogeneity of variance using the Kolmogorov–Smirnov and Levene tests, respectively.

With regard to the first goal, one multivariate analysis of variance (MANOVA) was conducted to assess the potential differences between the deaf pre-teens/teenagers and the control group on the SDQ four subscale scores. Another univariate analysis of variance (ANOVA) was run to investigate differences between the two groups on the SDQ total difficulties score. A Chi-square test was also performed to compare the number of pre-teens/teenagers (percentage pre-teens/teenagers) in the two groups that fell into different degrees of psychopathology.

In line with the second goal, ANOVAs were run to verify potential differences in anxiety, depression, resilience, and “time for sharing emotions” between the two groups of mothers. Chi-square tests were also performed to compare the number of mothers (percentage of mothers) in the two groups that fell into different degrees of anxiety and depression.

Pearson’s correlations were conducted to investigate the relationships between the mothers’ psychological features (i.e., GAD-7 scores, BDI-II scores, CD-RISC 25 scores, and time for sharing emotions) and psychological difficulties of the son/daughter (i.e., SDQ total difficulties scores) in the whole sample. The strength of the associations was considered as follows: ±0.10 represented a weak association, ±0.30 represented a medium association, and ±0.50 represented a high association [42].

## 3. Results

### 3.1. Psychological Well-Being for Pre-Teens/Teenagers with Cochlear Implants

For the SDQ mother’s version, the descriptive data and the results of the statistical comparisons are given in Table 3. The scores of the deaf teenagers were higher than those of normal-hearing teenagers across all subscales and in total difficulties score. Nevertheless, the MANOVA did not reveal any significant differences between the two groups of pre-teens/teenagers in any of the subscales and in the total difficulties score (Table 3). Table 4 shows the percentages of pre-teens/teenagers that fell into normality or different degrees of psychopathology on SDQ. The inspection of descriptive data revealed that a higher percentage of deaf pre-teens/teenagers with CIs fell into clinical risk categories for psychopathology, with as much as 33% classified as being at high risk. As indicated by the Chi-square analysis, this percentage was significantly higher in the group of deaf pre-teens/teenagers compared to their hearing peers (see Table 4).

### 3.2. Psychological Well-Being for Mothers of Pre-Teens/Teenagers with Cochlear Implants

The descriptive data for the GAD-7, BDI-II, and CD-RISC 25 total scores and the results of statistical comparisons are presented in Table 5. From the descriptive analysis, the scores reported by mothers on the GAD-7 were nearly identical. The time spent sharing emotions with their child also appeared to be similar in both groups of mothers. Instead, the scores reported by mothers of deaf pre-teens/teenagers on the BDI-II were higher compared to the control group, while the level of resilience appeared lower. Nevertheless, the two groups of mothers did not differ significantly from each other on any of these measures (Table 5). The two groups of mothers did not differ in the time for sharing emotions either (see Table 5). The Chi-square analysis also did not reveal differences in the percentage of mothers who fall into clinical risk categories for psychopathology (see Table 4).

### 3.3. Relationships Between Mother’s Psychological Features and Psychological Difficulties of the Son/Daughter

**Figure 1:**
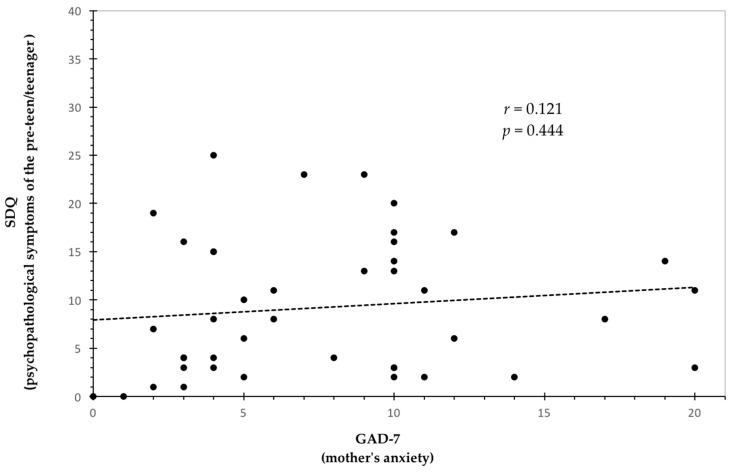

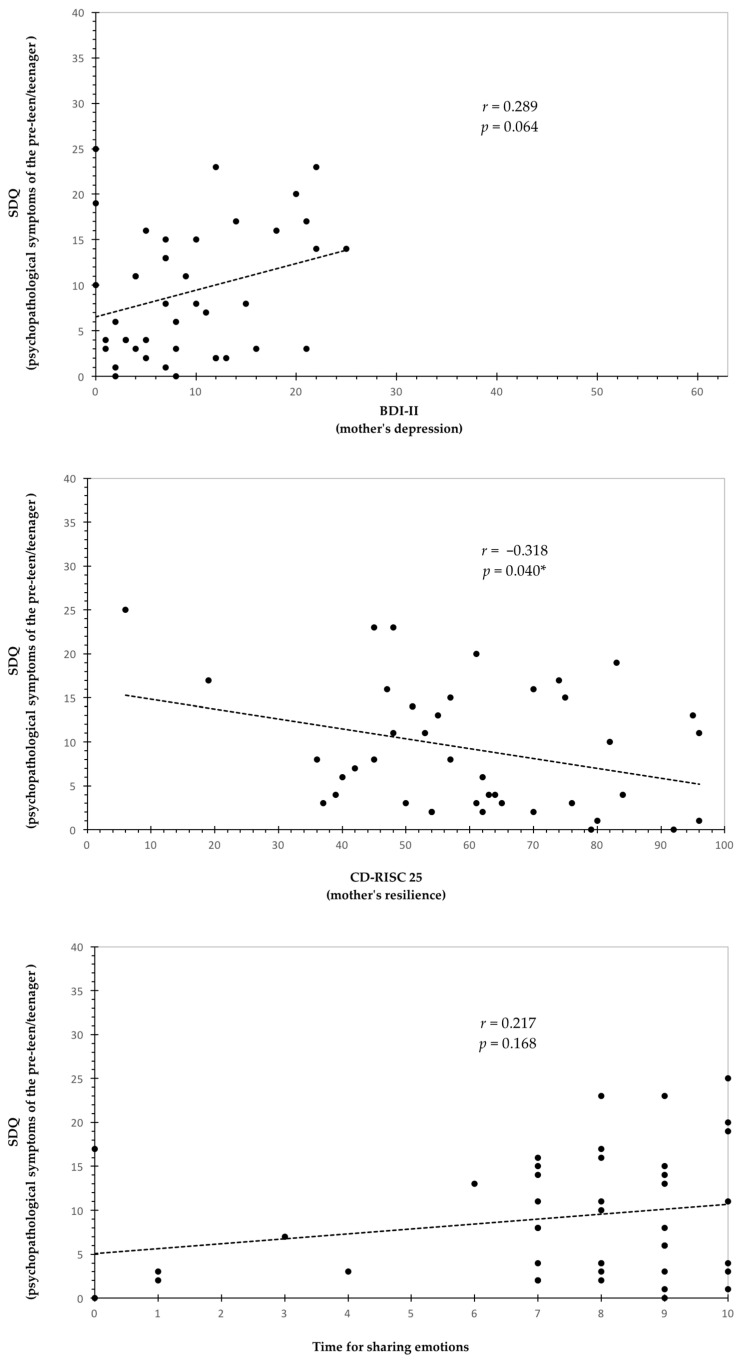
Correlation graphs between the four maternal variables (anxiety, depression, resilience, and time for sharing emotions) and the child’s psychological health (*r* and *p* values are reported) in the whole sample. * Significant correlation.

Table 6 shows the results of the correlations between the mother’s psychological features and the psychopathological symptoms of the son/daughter. The analysis indicated that maternal resilience (CD-RISC 25 scores) was significantly and negatively related to the psychopathological symptoms of the son/daughter (SDQ total difficulties scores). As shown in Table 6, the strength of this association was medium. Figure 1 provides a detailed representation of the relationship observed between the variables. No significant associations emerged between anxiety (GAD-7 scores) and depression symptoms (BDI-II scores) reported by the mothers and the psychological health of the son/daughter (see Table 6 and Figure 1). There were also no significant relationships between the time for sharing emotions and the psychopathological symptoms of the pre-teen/teenager (see Table 6 and Figure 1).

## 4. Discussion

In recent years, CIs have increasingly been performed in DHH children [43]. The effectiveness of CIs is related to both technological advancements and the worldwide spreading of audiological neonatal screening and early rehabilitation interventions [43]. Thus, the prompt use of CIs ensures, in a significant number of cases, a good auditory recovery and the development of language and learning skills similar to normal-hearing children and adolescents [43]. Interestingly, CI benefits have also been detected in children with deafness and additional disabilities [24]. Despite these advances, it is important to bear in mind that the CI restores peripheral hearing but does not eliminate the “deafness condition”, which may negatively impact the psychological well-being of the deaf child/adolescent and that of their families. This study aimed to analyze the psychological health of deaf pre-teens/teenagers with CIs, specifically relating it to that of their mothers.

As previously described, the recent literature on the psychological well-being of pre-teens/teenagers with CIs reveals conflicting data and is not extensive or exhaustive. Various psychological problems have been detected by some authors, such as oppositional behavior, hyperactivity-impulsivity, and social-adaptive skills [10,44,45]. Generally, children and adolescents with CIs seem to show more internalizing and externalizing symptoms than their normal-hearing peers [10]. From the descriptive analysis of pre-teens/teenagers in this study, it emerges that deaf participants achieve higher scores across all subscales and in the total difficulties score, reflecting greater emotional, behavioral, and social difficulties compared to their normal-hearing peers. Different from the literature reported above, the comparison of the scores on SDQ reported by the two groups of mothers in this study does not show significant differences in the SDQ subscales or the total difficulties score. Thus, the psychological condition of our deaf pre-teens/teenagers does not appear to be compromised; the pre-teens/teenagers with CIs appear to exhibit emotional and social well-being similar to their normal-hearing peers. As adolescence is a complex life stage marked by psychological processes such as identity formation, peer group inclusion, and self-esteem development, the consistent scores across all psychological SDQ subscales provide reassurance. These findings can be explained by the characteristics of the sample (i.e., pre-teens/teenagers without associated disabilities and early CIs), but also by the recent improvement in CI technical strategies. Nowadays, deaf adolescents may achieve good perceptual-auditory performance from infancy, becoming able to develop good linguistic and cognitive skills and, consequently, demonstrating positive emotional development, great social interactions, and a good QoL. Upon evaluating the psychosocial impact of CI use, evidence suggests that many recipients of CIs improve their communication skills in quiet environments and their ability to perceive loud sounds. The existing literature also indicates a development in self-confidence and social engagement among CI users [46]. Furthermore, a reduction in caregiver burden has been reported after CI surgery, underscoring the broader implications of caregiver support in society [47].

Although the data suggest positive psychological effects for pre-teens/teenagers with CI, it is important to interpret these findings with caution due to the small sample size and the descriptive analysis data of this study. From the analysis of qualitative data, additional findings emerge. Specifically, the percentage of pre-teens/teenagers falling below the SDQ clinical cut-offs shows that a significant number of subjects fall into a clinical range compared to the normal-hearing group. Therefore, although the scores reported in the SDQ by the mothers of the deaf pre-teens/teenagers are equivalent to those of the control group, a higher percentage of deaf pre-teens/teenagers are at higher risk of developing psychopathology disease. This result is not significantly different from that observed in earlier studies [48], which revealed a high prevalence of psychopathology in deaf adolescents. These results suggest the importance of conducting qualitative analyses in order to draw possible individual developmental trajectories. Data also highlight the importance of providing these patients with psychological support, along with auditory rehabilitation, especially during adolescence. As there is a lack of studies in this field and many of them are quantitative analysis, further qualitative investigations are needed in order to unmask problems emerging through the analysis of subjects and their clinical conditions.

In order to analyze whether the mothers of deaf pre-teens/teenagers with CIs and good auditory recovery might exhibit a psychological condition similar to or different from normal-hearing children, we assessed the mothers for symptoms of anxiety and depression, as well as for resilience, through standardized tests. We also focused on the frequency with which these mothers dedicate time to listening to their children’s emotions, thus enriching the study with qualitative data on the mother–child relationship. With regard to the psychological health of the mothers, different from previous studies [19,20], our analysis suggests that mothers of adolescents with CIs are not in a worse condition, neither in terms of anxiety nor depression, compared to mothers of the control group. The scores reported on the GAD-7 and BDI reflect conditions of mild anxiety and normality, respectively, in both groups. It is important to specify that our control group is consistent with the groups examined in other studies: the levels and prevalence of anxiety and depression observed in these mothers (mothers of hearing pre-teens/teenagers) are consistent with those reported by other researchers who investigated the psychological health of Italian mothers and women using the same questionnaires [47,48,49,50,51]. In line with the data observed in the sons/daughters, the good psychological status of mothers with deaf pre-teen/teenager may result from a better life perspective ensured by today’s technology and early CI intervention. As noted by some authors [14], the improvement in a deaf child’s cognitive, communicative, and social abilities is strongly related to the psychological health of the parents. Therefore, it can be hypothesized that where CIs ensure typical developmental trajectories, a significant improvement in the quality of life and psychological health of children, adolescents, and their mothers may be developed. Data from the descriptive analysis also show higher BDI-II scores in the mothers of deaf pre-teens/teenagers compared to the ones of normal-hearing peers. Therefore, it cannot be excluded that, with a larger sample, this difference might become significant. Additionally, data may vary considering that CIs do not eliminate the “deafness condition”, cannot be equated with the human ear, and may lead to different developmental outcomes. Analyzing the mothers’ time dedicated to discussing emotions with their son/daughter, the two groups of mothers do not differ. The literature emphasizes the emotional closeness of mothers as a determining factor in the health of the children [33,52]; however, this maternal feature was typically investigated using standardized questionnaires, which do not capture the frequency but rather only the presence/absence of this maternal behavior. Conversely, we asked participants to indicate how long these mothers and sons/daughters usually talk about the emotions felt. From our study, it emerged that the time dedicated to sharing emotions was approximately 5–15 min per week for both groups. We expected that this time would be greater in the mother–son/daughter with CI dyads, where the presence of deafness may make the pre-teen/teenager more vulnerable and, consequently, more in need of support and emotional closeness from adults [52,53]. Thus, we believe that this aspect requires greater attention in future research and should be studied in larger groups.

Psychological resources such as resilience are considered protective factors for mental health, alleviating psychopathological symptoms [28,54]. Resilience is not necessarily the complete absence of a stress response, but rather it refers to a mild and transient reaction to stress, which tends not to interfere with the ongoing ability to function [55]. As a matter of fact, even resilient individuals tend to experience at least some transient distress during or in the immediate aftermath of the traumatic event, although with a lower degree of severity. Observing the CD-RISC 25 scores, significant differences emerge between the two groups of mothers, and this appears to be a beneficial aspect for the mothers of deaf pre-teens/teenagers. However, from a descriptive analysis, both groups of mothers show lower resilience scores than expected. The CD-RISC 25 scores of both groups are similar to those reported by patients with psychopathological problems [40]. Testing the resilience in healthy subjects and patients with different diseases, including patients with Generalized Anxiety Disorders (GADs) and Post-Traumatic Stress Disorder (PTSD), Connor and Davidson [40] found CD-RISC 25 mean scores of 80.4 in the healthy group and always lower scores in the pathological group (GADs of 62.4 and PTSD of 52.8). Our study showed that mothers with deaf pre-teen/teenager and hearing pre-teen/teenager reported scores of 57.73 and 61.41, respectively. These scores are very close to those of patients with GADs. This result could be related to the fact that data were collected shortly after the end of the COVID-19 pandemic, a period in which the levels of resilience in Italian mothers were lower than in the past [34,56]. Low levels of resilience may represent a risk factor for mental health [51,56,57], especially in mothers with deaf pre-teens/teenagers who have to cope with this clinical condition. Although these mothers’ anxiety and depression scores appear normal, it is important to not underestimate the consequences of low resilience for other aspects of health over time.

Interesting results emerge from the investigation of the relationships between mothers’ psychological features and the psychological health of pre-teens/teenagers. A good level of maternal psychological health seems to have positive effects on the child’s development, also enhancing the parenting effectiveness in rehabilitation programs based on family involvement [58,59]. Therefore, the evaluation of the strength of these relationships is fundamental given the lack of studies in this clinical population. From these data, it also emerges that the absence or low levels of psychopathological symptoms, such as levels of anxiety or depression, do not influence the mental health of the son/daughter, but rather the mother’s positive features, namely resilience, show an effect. Specifically, the correlation analyses revealed a significant negative association between CD-RISC 25 scores and SDQ total difficulties scores. Therefore, resilient strategies of the mothers should be strengthened, particularly among mothers of pre-teens/teenagers with disabilities. In line with the “3 Cs” theoretical model of psychological resilience [60], the strategies of control (the belief in one’s ability to access personal resources to achieve short- and long-term goals), coherence (the inherent human desire to make sense of and find meaning in the world), and connectedness (the need for human contact and support) are identified as crucial resilience factors in the aftermath of stressful events [61]. These factors can provide psychological benefits to individuals [62]. Although preliminary, our results suggest a positive influence of maternal resilience strategies on the mental health of deaf sons/daughters.

With regard to the time that the mothers share with their son/daughter discussing their emotions, no significant associations emerged with the psychopathological symptoms of the pre-teen/teenager. The lack of correlation between these two variables seems to indicate that a higher level of symptoms in the pre-teen/teenager does not necessarily correspond to greater emotional closeness from the mother. In families with deaf pre-teens/teenagers, who may require more emotional support from the social context, in particular from their parents, this result can be seen as a potential factor of weakness, leading to possible negative long-term effects.

Although the present study provides new relevant insights, some limitations should be acknowledged. The primary limitation is the small sample size, which may have obscured the potential differences between groups and relationships in the analyzed variables. The results of the present pilot study should therefore be interpreted with caution. Furthermore, the generalizability of our findings should be carefully considered. Increasing the number of participants could also enable the performance of moderation and mediation analyses, thereby providing a more comprehensive understanding of the potentially indirect relationships among variables. For example, it may emerge that maternal anxiety and/or depression indirectly influence the mental health of the son/daughter, with a moderating or mediating effect of resilience. Moreover, longitudinal studies and regression analyses examining the effects of maternal variables on the psychological health of deaf teenagers would be highly interesting. Future research should further explore the complexities of these relationships. Despite this limitation, the results of this pilot investigation appear sufficient to suggest the associations between the psychological features of the mothers and the psychological health of deaf pre-teens/teenagers. Another limitation is the absence of self-reported assessments from the pre-teens/teenagers regarding their psychological health and quality of life. Future research should aim to assess whether discrepancies exist between maternal reports and pre-teens/teenagers’ self-reports concerning, for instance, emotional and social health. Moreover, although this study includes only pre-teens/teenagers without neurodevelopmental disorders, it lacks direct measures that accurately assess language performance and academic learning. The broad heterogeneity characterizing this clinical population in terms of linguistic and academic outcomes is well known [63,64]. It should be noted that the results obtained here might differ significantly when focusing on deaf children and adolescents who exhibit language and academic learning difficulties [52]. Thus, future studies should focus on the psychological health of those children and adolescents who, despite receiving CIs, experience language and learning challenges [64].

Additionally, in order to gain a more comprehensive knowledge of the psychological consequences of deafness and to understand whether these different conditions or choices may influence the psychological health and quality of life of these adolescents and their families differently, it would be necessary to compare deaf teenagers with CIs who are non-signing, those with CIs who are signing, and deaf teenagers who are only signing. The comparison between deaf children and adolescents with CIs and their peers with hearing aids could also help to better understand the impact that the choice of cochlear implantation may have on the quality of life of these patients and provide additional insights useful to the guidance of clinical practice. It would be desirable for research to proceed in these directions.

## 5. Conclusions

This pilot study focuses on deaf pre-teens and teenagers with CIs who achieved good auditory recovery and did not exhibit associated cognitive or communicative issues. It also investigates whether this condition positively influences their psychological health. Moreover, the study examines the mental health of their mothers and explores their relationships with their deaf children. Despite the limited sample size, our data suggest that pre-teens/teenagers with CIs experience good psychological health compared to their normal-hearing peers. The same could be applied to the mothers of these adolescents, who do not appear to exhibit higher levels of anxiety and depressive symptoms compared to mothers in the control group. The qualitative analysis reveals noteworthy findings, highlighting that a higher percentage of pre-teens/teenagers with CIs remain at risk for developing psychopathology. These data underscore the necessity of both identifying risk factors that may lead to negative psychological outcomes early and providing targeted psychological support. Mothers’ resilience scores are lower than expected too (i.e., lower compared to those reported in other studies), reflecting a vulnerability in coping with parenting demands and their child’s deafness. Importantly, maternal resilience appears to be associated with the psychological health of the pre-teen/teenager. From this study, the importance of fostering supportive family environments and enhancing family-centered care for pre-teens/teenagers with CIs emerges. Clinical follow-ups with deaf children and adolescents should not be limited to the evaluation of the CI function and auditory performance, but it is also essential to incorporate psychological counseling services where these patients and their families can be supported in facing new challenges. Furthermore, it is important to promote psycho-educational interventions for parents and educators, offering concrete guidance on how to support the emotional development of these children and adolescents. These interventions should also highlight the necessity of dedicating time to emotional dialogue with these pre-teens/teenagers and consider the emotional closeness of the adult as a potential protective factor for the mental health of these patients. Further research is needed in order to shed light on the remaining unsolved questions.

## Figures and Tables

**Table 1:** Characteristics of the group of deaf pre-teens/teenagers with CIs and the group of hearing peers.

	Deaf Pre-Teens/Teenagers with CI (n = 15)	Hearing Pre-Teens/Teenagers (n = 27)
Chronological age	M = 13.16 years (SD = 2.27; range 10.42–16.83)	M = 13.40 (SD = 1.21; range 11.67–16.83)
Sex	8 males (53.3%), 7 females (46.7%)	11 males (40.7%), 16 females (59.3%)
Hearing Aids (age at first amplification)	Before 24 months	-
Speech therapy (age at initiation)	Before 24 months	-
Type of speech therapy	Oral *	-
Age at CI surgery	Before 3 years	-
Type of school	Traditional school education	Traditional school education
Grade of school	Middle and high schools	Middle and high schools
Mathers’ age	M = 43.53 (SD = 5.22; range 37–54)	M = 46.96 (SD = 5.26; range 33–56)
Maternal educational level	4 low level (26.7%), 11 high level (73.3%)	4 low level (14.8%), 23 low level (85.2%)

* Non-use of sign language.

**Table 2:** Types of responses to the ad hoc question concerning the “time for sharing emotions” and the respective numerical values assigned to the responses.

Type of Responses	Numerical Value
Almost never	0
5–15 min per year	1
30 min per year	2
5–15 min per month	3
30 min per month	4
5–15 min every 15 days	5
30 min every 15 days	6
5–15 min per week	7
30 min per week	8
5–15 min per day	9
30 or more minutes per day	10

**Table 3:** Descriptive data (mean and standard deviation) for the SDQ scores and results of statistical comparisons (MANOVA and ANOVA) between deaf pre-teens/teenagers with CIs and hearing pre-teens/teenagers.

SDQ Scores	Deaf Pre-Teens/Teenagers with CI (n = 15)		Hearing Pre-Teens/Teenagers (n = 27)		MANOVA		
	M (SD)	Range	M (SD)	Range	*F*	*p*	*d*
Emotional	3.27 (2.81)	0–9	2.22 (2.42)	0–9	1.596	0.214	0.40
Conduct	2.53 (1.96)	0–6	1.56 (1.69)	0–6	2.872	0.098	0.53
Hyperactivity	3.47 (1.88)	0–6	2.15 (2.33)	0–7	3.508	0.068	0.62
Social	2.60 (2.50)	0–8	1.81 (1.69)	0–6	1.471	0.232	0.37
					ANOVA		
	M (SD)	range	M (SD)	range	*F*	*p*	*d*
Total difficulties score	11.87 (7.61)	1–23	7.74 (6.43)	0–25	3.48	0.069	0.59

**Table 4:** Percentages of pre-teens/teenagers that fell into normality or into different degrees of psychopathology on SDQ and percentages of mothers that fell into normality or into different degrees of psychopathology on GAD7 and BDI-II.

	Deaf Pre-Teens/Teenagers with CI (n = 15)	Hearing Pre-Teens/Teenagers (n = 27)	Chi-Square		
	N (%)	N (%)	*χ* ^2^	*p*	*phi*
SDQ Total difficulties score			4.10	**0.043**	0.09
Not risk of psychopathology	8 (53.3%)	21 (77.8%)	-	-	-
Slight risk of psychopathology	2 (13.3%)	4 (14.8%)	-	-	-
High risk of psychopathology	5 (33.4%)	2 (7.4%)	-	-	-
GAD-7 scores			0.059	0.808	0.09
Normality	5 (33.3%)	11 (40.7%)	-	-	-
Mild anxiety	6 (40.1%)	3 (11.1%)	-	-	-
Moderate anxiety	2 (13.3%)	11 (40.7%)	-	-	-
Severe anxiety	2 (13.3%)	2 (7.5%)	-	-	-
BDI-II scores			3.43	0.064	0.10
Normality	7 (46.7%)	18 (66.7%)	-	-	-
Mild depression	3 (20.0%)	7 (25.9%)	-	-	-
Moderate depression	5 (33.3%)	2 (7.4%)	-	-	-
Severe depression	0 (0%)	0 (%)	-	-	-

Significant results are in bold.

**Table 5:** Descriptive data (mean and standard deviation) for GAD-7, BDI-II, CD-RISC 25, and “time for sharing emotions” scores and results of statistical comparisons (ANOVAs) between mothers of deaf pre-teens/teenagers and mothers of hearing adolescents.

	Mothers of Deaf Pre-Teens/Teenagers with CI (n = 15)		Mothers of Hearing Pre-Teens/Teenagers (n = 27)		ANOVAs		
	M (SD)	Range	M (SD)	Range	*F*	*p*	*d*
GAD-7	7.27 (5.68)	2–20	7.89 (4.91)	0–20	0.138	0.712	0.12
BDI-II	10.73 (8.80)	0–22	8.22 (5.67)	0–25	1.268	0.267	0.34
CD-RISC 25	57.73 (21.22)	19–96	61.41 (19.51)	6–96	0.321	0.574	0.18
Time for sharing emotions	7.27 (2.66)	0–10	7.44 (2.79)	0–10	0.040	0.842	0.06

**Table 6:** Pearson’s correlation analysis between the mother’s psychological features (GAD-7 scores, BDI-II scores, CD-RISC 25 scores, and time for sharing emotions) and the child’s psychopathological symptoms (SDQ total difficulties scores) in the whole sample.

Maternal Variables	Pearson’s Correlations with the SDQ Total Difficulties Scores (*r*)
GAD-7 scores (anxiety)	0.121
BDI-II scores (depression)	0.289
CD-RISC 25 scores (resilience)	−0.318 *
Time for sharing emotions	0.217

Significant results are in bold. * *p* < 0.05

## Data Availability

The data presented in this study are available on request from the corresponding author. The data are not publicly available due to privacy reasons.
